# Proteomic analysis identifies MMP-9, DJ-1 and A1BG as overexpressed proteins in pancreatic juice from pancreatic ductal adenocarcinoma patients

**DOI:** 10.1186/1471-2407-8-241

**Published:** 2008-08-16

**Authors:** Mei Tian, Ya-Zhou Cui, Guan-Hua Song, Mei-Juan Zong, Xiao-Yan Zhou, Yu Chen, Jin-Xiang Han

**Affiliations:** 1 Key Laboratory of Ministry of Health for Biotech-Drug, Key Laboratory for Modern Medicine and Technology of Shandong Province, Shandong Medicinal Biotechnology Center, Shandong Academy of Medical Sciences, Jinan 250062, PR China

## Abstract

**Background:**

There is an urgent need to discover more sensitive and specific biomarkers to improve early diagnosis and screen high-risk patients for pancreatic ductal adenocarcinoma (PDAC). Pancreatic juice is an ideal specimen for PDAC biomarkers discovery, because it is an exceptionally rich source of proteins released from pancreatic cancer cells.

**Methods:**

To identify novel potential biomarkers for PDAC from pancreatic juice, we carried out difference gel electrophoresis (DIGE) and tandem mass spectrometry (MS/MS) to compare the pancreatic juice profiling from 9 PDAC patients and 9 cancer-free controls. Of the identified differently expressed proteins, three up-regulated proteins in pancreatic cancer juice, matrix metalloproteinase-9 (MMP-9), oncogene DJ1 (DJ-1) and alpha-1B-glycoprotein precursor (A1BG), were selected for validation by Western blot and immunohistochemistry. Serum MMP-9 levels were also detected by enzyme linked immunosorbent assay (ELISA).

**Results:**

Fourteen proteins were up-regulated and ten proteins were down-regulated in cancerous pancreatic juice compared with cancer-free controls. Increased MMP-9, DJ-1 and A1BG expression in cancerous pancreatic juice were confirmed by Western blot. Immunohistochemical study showed MMP-9, DJ-1 and A1BG positively expressed in 82.4%, 72.5% and 86.3% of pancreatic cancer tissues, significantly higher than that in normal pancreas tissues. Up-regulation of DJ-1 was associated with better differentiation (p < 0.05). Serum MMP-9 levels were significantly higher in PDAC (255.14 ng/ml) than those in chronic pancreatitis (210.22 ng/ml, p = 0.009) and healthy control (203.77 ng/ml, p = 0.027).

**Conclusion:**

The present proteome analysis revealed MMP-9, DJ-1 and A1BG proteins as elevated in pancreatic juice from PDAC, which suggest their further utility in PDAC diagnosis and screening. This is the first time A1BG was identified as a potential biomarker in pancreatic cancer associated samples. The measurement of serum MMP-9 might be clinically useful for PDAC diagnosis.

## Background

Pancreatic ductal adenocarcinoma (PDAC) is the fourth or fifth most common cause of cancer-related mortality worldwide. Because of late presentation and rapid aggressiveness, most PDAC cases are diagnosed at late stage, and its prognosis is accordingly poor. So detection of PDAC at an early disease stage is critical for successful clinical therapy. Carbohydrate antigen (CA) 19-9 is the most commonly used tumor marker for PDAC, but it lacks satisfactory sensitivity and specificity, especially in early diagnosis [1,2]. There is an urgent need to discover more sensitive and specific biomarkers to improve early diagnosis and screen high-risk patients for PDAC [3].

The proteomic approach is a powerful tool to identify novel biomarkers or therapeutic targets from cancer-associated samples. Pancreatic juice is an ideal specimen in proteomic studies, because it is an exceptionally rich source of proteins which are released from pancreatic cells in the physiological state and in pathological conditions [4]. It is reasonable that biomarkers identified in pancreatic juice could subsequently be measured in more accessible specimens such as serum. Therefore, mining pancreatic juice proteome might help to identify novel protein markers for pancreatic diseases such as pancreatic cancer.

Recently, many efforts have been made to analyze pancreatic juice by proteomic methods. Rosty et al. [5] identified hepatocarcinoma-intestine-pancreas/pancreatitis-associated protein I (HIP/PAP-I) as a biomarker for PDAC by surface enhanced laser desorption ionization (SELDI) techonology. Chen et al. [6] used isotope-code affinity tag (ICAT) technology to compare the pancreatic juice protein profiling from pancreatitis patients and normal controls. Gronborg et al. [3] used one-dimensional electrophoresis combined with liquid chromatography tandem mass spectrometry (1-DE-LC-MS/MS) to identify a total of 170 unique proteins in pancreatic juices from 3 cases of PDAC patients, and confirmed PAP-2 as a novel marker for PDAC.

In the current study, we characterized the pancreatic juice protein profiling in PDAC compared with cancer-free controls using difference gel electrophoresis (DIGE) and tandem mass spectrometry (MS/MS), and identified a number of novel protein markers in pancreatic juice which might be a promising target for pancreatic cancer diagnosis and screening.

## Methods

### Patients and samples

The study was approved by the Ethical   Commitee of Shandong Academy of Medical Science. Fresh pancreatic juice samples were collected with informed consent from 9 PDAC patients and 9 cancer-free controls undergoing endoscopic retrograde cholangiopancreatography (ERCP) in Qilu Hospital and Qianfoshan Hospital of Shandong University. Clinical data of the patients included were summarized in Table 1. After collection, the pancreatic juice samples were centrifuged at 10,000 rpm for 20 min at 4°C and supernatant of each was aliquoted and stored at -80°C until used.

**Table 1:** Clinicopathological data of PDAC patients and cancer-free controls in proteomic analysis

No. of samples	Sex	Age	Histology*	Tumor location	Metastases
PDAC					
1	female	55	Moderately differentiated ductal adenocarcinoma	Tail of pancreas	Yes
2	male	65	Well differentiated ductal adenocarcinoma	Head of pancreas	No
3	male	38	Well differentiated ductal adenocarcinoma	Head of pancreas	Yes
4	male	57	Moderately differentiated ductal adenocarcinoma	Body of pancreas	Yes
5	female	53	Poorly differentiated ductal adenocarcinoma	Head of pancreas	No
6	male	59	Moderately differentiated ductal adenocarcinoma	Head of pancreas	Yes
7	female	44	Moderately differentiated ductal adenocarcinoma	Tail of pancreas	Yes
8	female	51	Well differentiated ductal adenocarcinoma	Head of pancreas	No
9	male	48	Moderately differentiated ductal adenocarcinoma	Tail of pancreas	No

Cancer-free			Clinical diagnosis		

1	male	53	Chronic pancreatitis		
2	female	66	benign cystic neoplasm of pancreas		
3	male	49	Gallstone pancreatitis		
4	male	50	Chronic pancreatitis		
5	female	52	benign cystic neoplasm of pancreas		
6	female	57	benign cystic neoplasm of pancreas		
7	male	46	Chronic pancreatitis		
8	female	47	cystic fibrosis of pancreas		
9	male	60	Chronic pancreatitis		

* PDAC differentiation was classified according to the WHO standard (2nd edition, 1996).

### Pancreatic juice protein extraction

Pancreatic juice samples were first precipitated with acetone. Briefly, 1.2 mL cold acetone (Fluca) was added to 300 μL pancreatic juice and kept at -20°C for 2 hours, then centrifuged at 13,000 rpm for 10 min at 4°C. Supernatant was discarded and pellet was dissolved in 500 μL of lysis buffer containing 30 mM Tris, 8 M urea, 2 M thiourea, 2% 3-[(3-cholamidopropyl) dimethylammonio]-1-propanesulfonate (CHAPS), 18 mM dithiothreitol (DTT) and 0.5% IPG Buffer (GE Healthcare). The mixture was then centrifuged at 12,000 rpm for 10 min at 4°C, and the supernatant was collected and stored at -80°C. Protein concentration was determined using a 2-D Quant Kit according to the manufacturer's instruction (GE Healthcare).

### Two-dimensional gel electrophoresis (2-DE)

Cancerous or control pancreatic juice protein extracts were first pooled for traditional 2-DE. Two hundred micrograms of each pooled protein sample was diluted in 450 μL rehydration buffer (7 M urea, 2 M thiourea, 4% CHAPS, 0.5% IPG buffer, 0.002% bromophenol blue). Isoelectric focusing was performed on Ettan IPGphor (GE Healthcare) with 24 cm IPG strips (pH 3–10 NL, GE Healthcare). The IPG strips were first rehydrated at 30 V for 12 hours, then focused at 500 V for 1 hour, 1,000 V for 1 hour, and maintained at 8,000 V until a total of 65,000 Vhr was arrived. After isoelectric focusing, the strips were equilibrated with 0.375 M Tris-HCl (pH 8.8), 6 M urea, 20% glycerol, 2% sodium dodecyl sulfate (SDS), and 0.2% bromophenol blue. IEF strips were first treated with 130 mM DTT for 10 min, followed by 135 mM iodoacetamide for 10 min with constant shaking. The equilibrated strips were transferred to 12.5% SDS polyacrylamide gel electrophoresis (SDS-PAGE) on Ettan DALT twelve system (GE Healthcare) with constant power (0.2 W/gel, 1 hour; 1.7 W/gel, 4.5 hours) at 20°C. All gels were stained with Coomassie blue R350 (GE Healthcare), and scanned using a PowerLook 2100 XL scanner system (Umax USA).

### Two-dimensional difference gel electrophoresis (DIGE)

Fifty micrograms of each of cancerous and cancer-free control pancreatic juice protein extracts was minimally labeled with 400 pmol Cy3 or 400 pmol Cy5 fluorescent dye (GE Healthcare). An internal standard pool generated by combining equal amounts of extracts from all the samples was labeled with 400 pmol Cy2. The labeling reaction was carried out in the dark on ice for 30 min, and quenched with 10 mM lysine for 10 min. 2-DE was performed as described above, except that low-fluorescent glass plates were used. The Cy2, Cy3, and Cy5-labeled images were acquired on a Typhoon Trio scanner (GE Healthcare) at the excitation/emission of 488/520 nm, 532/580 nm and 633/670 nm, respectively.

### Trypsin digestion and MALDI-TOF-MS and MS/MS

Spots of interest were excised from gels stained by Coomassie Blue R350, and were digested with sequencing grade modified porcine trypsin (Promega) as described previously [7]. Subsequent protein identification was carried out on the ABI 4700 Proteomic Analyzer MALDI-TOF-MS/MS mass spectrometry (Applied Biosystems, USA) on a reflective mode. Peptide mass fingerprint (PMF) was acquired between 800–3500 Da. The strongest five peaks from PMF were selected to obtain MS/MS spectra. PMF and MS/MS data were then searched against a human subset of the Swiss-Prot database using GPS explorer software (Applied Biosystems, USA).

### Western blot

Thirty micrograms of pancreatic juice protein extracts from 6 PDAC and 6 cancer-free controls were used for SDS-PAGE. The separated proteins were transferred to nitrocellulose membranes (GE Healthcare). The membranes were blocked for 1 hour and incubated with anti-DJ-1 antibody (1:1000 dilutions, MBL international corporation, MA), anti-MMP-9 antibody (1:1000 dilutions, Santa Cruz Biotechnology) and anti-A1BG antibody (1:1000 dilutions, Aviva System Biology, San Diego, CA) overnight at 4°C. After peroxidase-conjugated secondary antibody was added, proteins were detected using an ECL Plus kit (GE Healthcare).

### Immunohistochemical analysis (IHC)

To study the tissue compartment for DJ-1, MMP-9 and A1BG, a tissue array (Chaoying Biotechnology co. Xian, China) containing 51 cases of PDAC and 8 adjacent normal pancreas tissues was used in immunohistochemical analysis. Clinicopathological data of the tissue array were summarized (Table 2). Paraffin-embedded tissue array slides were processed for antigen retrieval using microwave heating in citrate buffer, and immunohistochemically stained with the same antibodies used in Western blot analysis at 1:100 dilution. Visualization was performed using ABC kit according to the manufacturer's recommendation (Zhongshan Bio co., Beijing, China).

**Table 2:** Data of tissue array included in immunohistochemical analysis

No. of samples	Age	Sex	Histology	Grade
1	64	Male	Moderately differentiated ductal adenocarcinoma	II
2	64	Male	Poorly differentiated ductal adenocarcinoma	III
3	64	Male	Moderately differentiated ductal adenocarcinoma	II
4	62	Female	Poorly differentiated ductal adenocarcinoma	III
5	62	Female	Poorly differentiated ductal adenocarcinoma	III
6	62	Female	Poorly differentiated ductal adenocarcinoma	III
7	12	Female	Normal pancreatic tissue	-
8	12	Female	Normal pancreatic tissue	-
9	12	Female	Normal pancreatic tissue	-
10	49	Male	Poorly differentiated ductal adenocarcinoma	III
11	49	Male	Moderately differentiated ductal adenocarcinoma	II
12	49	Male	Moderately differentiated ductal adenocarcinoma	II
13	69	Female	Moderately differentiated carcinoma	II
14	69	Female	Moderately differentiated carcinoma	II
15	69	Female	Well differentiated Mucinous adenocarcinoma	I
16	73	Female	Poorly differentiated ductal adenocarcinoma	III
17	73	Female	Poorly differentiated ductal adenocarcinoma	III
18	73	Female	Poorly differentiated carcinoma	III
19	49	Male	Moderately differentiated ductal adenocarcinoma	II
20	49	Male	Moderately differentiated ductal adenocarcinoma	II
21	49	Male	Moderately differentiated ductal adenocarcinoma	II
22	67	Male	Poorly differentiated ductal adenocarcinoma	III
23	67	Male	Poorly differentiated ductal adenocarcinoma	III
24	67	Male	Poorly differentiated ductal adenocarcinoma	III
25	68	Female	Moderately differentiated ductal adenocarcinoma	II
26	68	Female	Moderately differentiated ductal adenocarcinoma	II
27	68	Female	Moderately differentiated ductal adenocarcinoma	II
28	51	Female	Moderately differentiated ductal adenocarcinoma	II
29	51	Female	Poorly differentiated ductal adenocarcinoma	III
30	51	Female	Poorly differentiated ductal adenocarcinoma	III
31	45	Male	Well differentiated ductal adenocarcinoma	I
32	45	Male	Well differentiated ductal adenocarcinoma	I
33	45	Male	Well differentiated ductal adenocarcinoma	I
34	60	Male	Poorly differentiated ductal adenocarcinoma	III
35	60	Male	Poorly differentiated ductal adenocarcinoma	III
36	60	Male	Moderately differentiated ductal adenocarcinoma	II
37	65	Male	Poorly differentiated ductal adenocarcinoma	III
38	65	Male	Poorly differentiated ductal adenocarcinoma	III
39	65	Male	Moderately differentiated ductal adenocarcinoma	II
40	55	Male	Poorly differentiated ductal adenocarcinoma	III
41	55	Male	Poorly differentiated ductal adenocarcinoma	III
42	55	Male	Poorly differentiated ductal adenocarcinoma	III
43	70	Male	Well differentiated ductal adenocarcinoma	I
44	70	Male	Moderately differentiated ductal adenocarcinoma	II
45	70	Male	Well differentiated ductal adenocarcinoma	I
46	30	Male	Normal pancreatic tissue	-
47	30	Male	Normal pancreatic tissue	-
48	51	Female	Well differentiated ductal adenocarcinoma	I
49	51	Female	Well differentiated ductal adenocarcinoma	I
50	51	Female	Well differentiated ductal adenocarcinoma	I
51	62	Female	Moderately differentiated papillary carcinoma	II
52	62	Female	Moderately differentiated ductal adenocarcinoma	II
53	62	Female	Moderately differentiated ductal adenocarcinoma	II
54	50	Male	Poorly differentiated ductal adenocarcinoma	III
55	50	Male	Poorly differentiated ductal adenocarcinoma	III
56	50	Male	Poorly differentiated ductal adenocarcinoma	III
57	33	Female	Normal pancreatic tissue	-
58	14	Female	Normal pancreatic tissue	-
59	38	Female	Normal pancreatic tissue	-

### Enzyme linked immunosorbent assay (ELISA)

Serum from 8 PDAC patients, 9 chronic pancreatitis patients and 8 healthy controls not included in the proteomic analysis were collected for MMP-9 ELISA analysis. Serum levels of human MMP-9 were analyzed with a commercially available kit (Biotrak) according to the manufacturer's recommendation. Serum samples were diluted at 1:100; all assays were done in duplicate. The sensitivity limit of MMP-9 ELISA was 0.08 ng/ml.

### Data analysis

DIGE images were analyzed by ImageMaster 6.0 DIGE-enable software (GE Healthcare). The best internal standard image was assigned as the master reference. The protein spots on the remaining internal standard images were matched to the master reference to ensure that the same protein spots were compared between gels. Spot intensity was normalized by dividing each Cy3 or Cy5 spot volume with the corresponding Cy2 (internal standard) spot volume. Statistical analyses were performed using SPSS 13.0 software. The relationships with DJ-1, MMP-9 and A1BG expression and clinicopathological parameters were analyzed using Chi-square or Fisher's exact tests. The differences of MMP-9 levels in serum among the various groups were analyzed using independent-samples t-test. p < 0.05 was considered to be statically significant.

## Results

### Differentially expressed Proteins in pancreatic juice from PDAC and cancer-free controls

**Figure 1:**
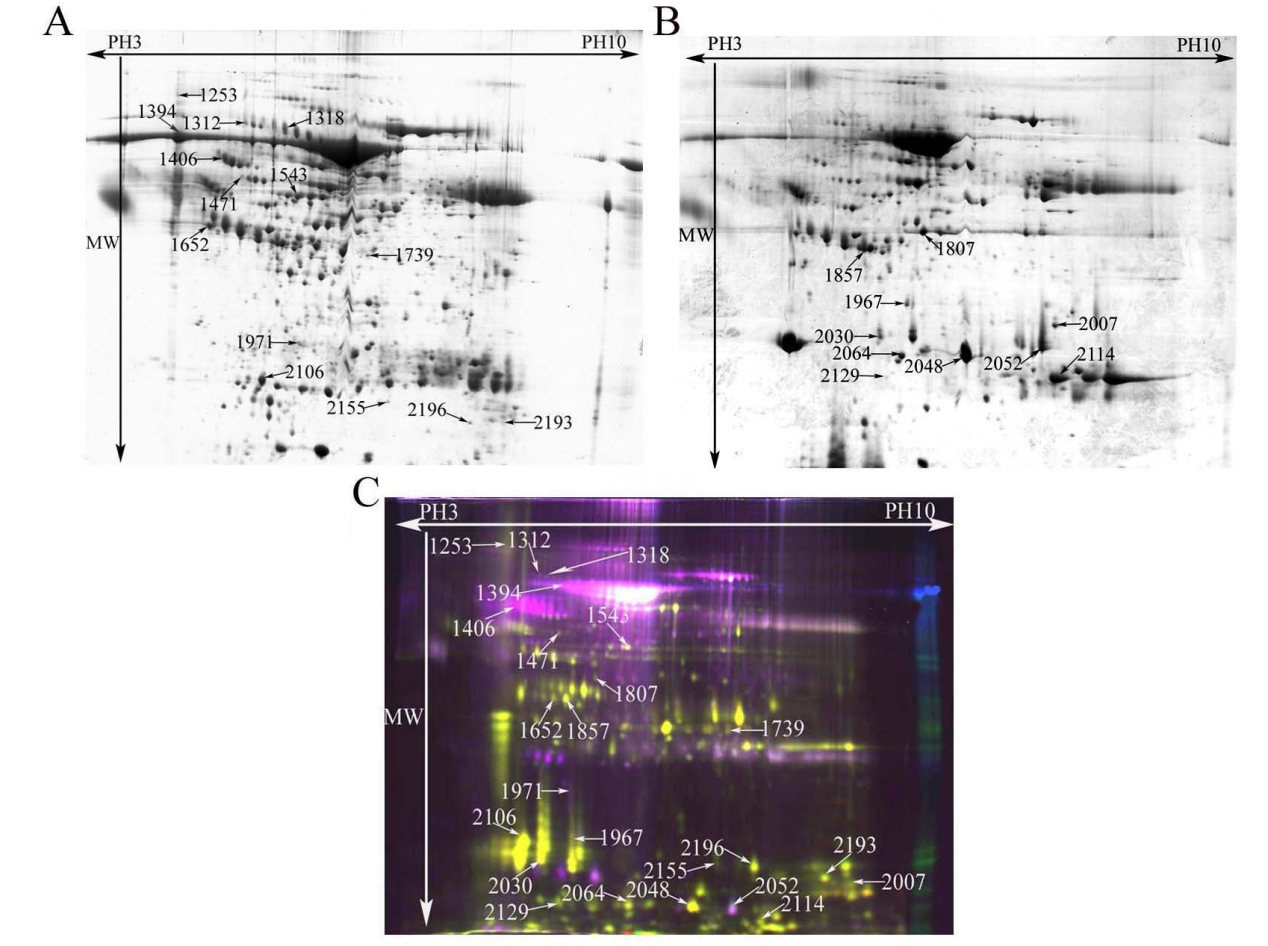
**Representative gel images of proteins extracted from pancreatic cancer juice and cancer-free controls juice**. Representative 2-DE gel images of pancreatic juice proteins from PDAC (A) and cancer-free controls (B), and representative DIGE overlay image (C). Labeled spots are significantly up-regulated proteins in pancreatic cancer juice (A), in cancer-free controls juice (B) and a total of 24 differentially expressed protein spots (C).

**Figure 2:**
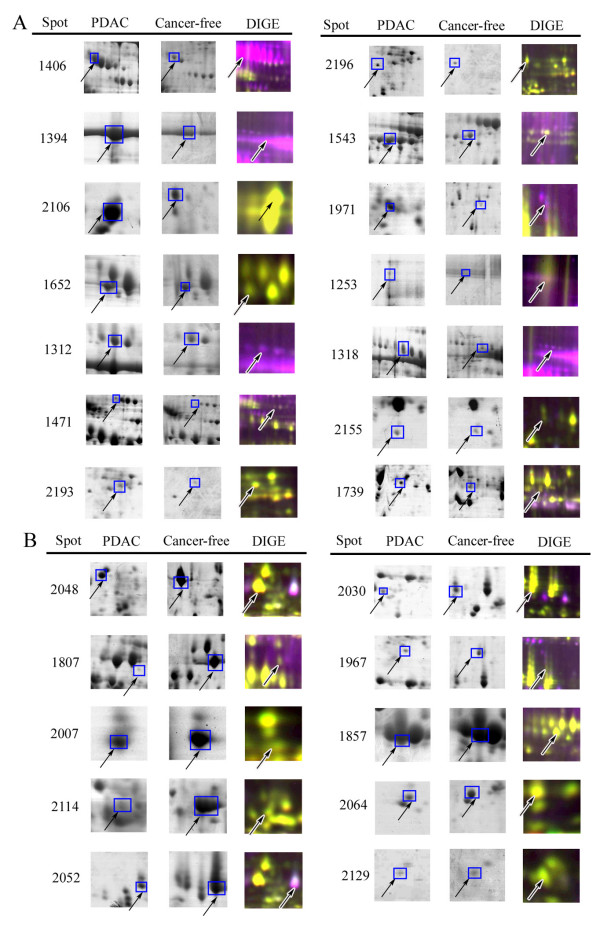
**Individual differentially expressed protein spots in pancreatic cancer juice and cancer-free controls juice**. Selected areas of 14 up-regulated (A) and 10 down-regulated (B) spots and their corresponding DIGE images.

Given the limited amount of pancreatic juice sample available, we first made sample pools of all cancerous and cancer-free control pancreatic juice protein extracts separately to identify the differently expressed proteins by traditional 2-DE. Each pool was repeated three times. Coomassie blue R350 staining was applied to visualize the protein spots, because of its compatibility with protein identification by MS. Moreover, we carried out DIGE on each individual sample to verify differential protein expression found by the traditional 2-DE. Most spot changes in DIGE analysis were consistent with those found in Coomassie blue stained 2-DE analysis (Figure 1). Twenty four proteins with more than two-fold expression change between pancreatic cancer juice and cancer-free pancreatic juice were identified (Table 3). Fourteen proteins were significantly up-regulated and ten proteins were significantly down-regulated in pancreatic cancer juice compared with the cancer-free controls (Figure 2). Three up-regulated proteins MMP-9, DJ-1 and A1BG were further confirmed by Western blot and immunohistochemistry.

**Table 3:** Differentially expressed proteins in pancreatic juice from PDAC patients and cancer-free controls (with and without pancreatitis)

Spot position	Protein Name	MW(Da)	PI	Mascot PMF Score	Sequence Coverage (%)	Protein Species Score (C.I.%)	Fold change	
							
							PDAC/Cancer-free controls	Pancreatitis/No pancreatitis controls
1406	Alpha-1-antitrypsin precursor	46737	5.37	298	39.788	100	3.43	-1.72
1394	Serum albumin precursor	69367	5.92	491	73.493	100	2.67	-1.27
2106	Apolipoprotein A-I precursor	30778	5.56	289	56.054	100	4.83	1.30
1652	Glutathione S-transferase P	32311	5.43	90	68.249	99.998	3.84	-1.36
1312	Alpha-1B-glycoprotein precursor	54273	5.58	124	33.863	100	2.39	-1.37
1471	Vitamin D-binding protein precursor	52964	5.40	300	60.622	100	5.60	-2.03
2193	Cationic antimicrobial protein	26886	9.75	127	38.512	99.997	2.73	-3.90
2196	Superoxide dismutase [Mn], mitochondrial precursor	24722	8.35	109	36.524	100	2.54	2.12
1543	Serotransferrin precursor	77050	6.81	519	76.674	100	5.09	1.03
1971	Ig lambda chain V-IV region Hil	11517	6.04	102	49.056	100	2.66	1.44
1253	matrix metalloproteinase-9	78427	5.69	214	32.458	100	3.67	-1.03
1318	Hemopexin precursor	51676	6.55	213	42.289	100	3.23	1.06
2155	Oncogene DJ1	19891	6.33	114	15.878	100	2.59	-1.25
1739	Fibrinogen beta chain precursor	55928	8.54	247	53.067	100	4.15	-2.56
2048	Trypsin-1 precursor	26558	6.08	121	35.176	100	-2.64	2.80
1807	Carboxypeptidase A2 precursor	46828	5.68	208	52.449	100	-13.00	1.41
2007	Complement C4-B precursor	192793	6.73	123	31.488	100	-2.10	-1.50
2114	Chymotrypsinogen B precursor	27870	6.79	146	36.051	100	-2.43	1.30
2052	Elastase-3A precursor	29475	6.43	143	35.156	100	-5.71	-1.80
2030	Elastase-3B precursor	29293	5.65	157	42.945	100	-2.30	1.12
1967	Fibrinogen-like protein 1 precursor	36392	5.58	196	57.347	100	-2.71	-1.51
1857	Haptoglobin precursor	45205	6.13	163	45.013	100	-2.00	-1.72
2064	Trypsin-3 precursor	32499	7.46	120	27.118	100	-2.82	3.20
2129	Complement C3 precursor	187148	6.02	248	54.849	100	-3.78	4.04

### Western blot analysis of MMP-9, DJ-1 and A1BG

**Figure 3:**
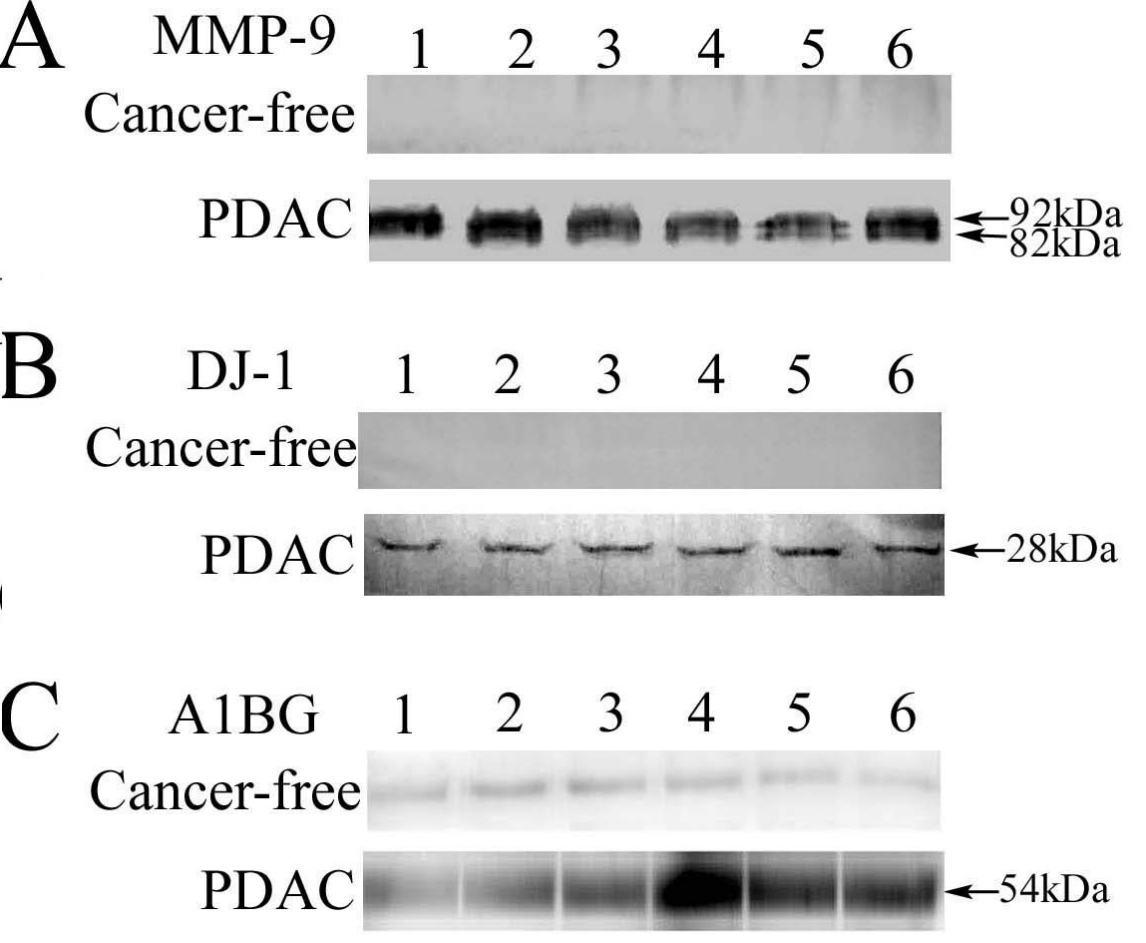
**MMP-9, DJ-1 and A1BG expression analysis by Western blot**. Increased MMP-9 (A), DJ-1 (B) and A1BG (C) were detected in the cancerous juice samples compared with cancer-free pancreatic juice samples; MMP-9 expression evinced both 92 kDa and 82 kDa bands, corresponding to the latent and activated forms of MMP-9, respectively.

As seen in Figure 3, compared with cancer-free controls, increased MMP-9, DJ-1 and A1BG expression in cancerous pancreatic juice were detected by Western blot. In addition, cancerous pancreatic juice with MMP-9 expression evinced both 92 kDa and 82 kDa bands, corresponding to the latent and activated forms of MMP-9, respectively.

### Immunohistochemical validation of MMP-9, DJ-1 and A1BG

**Figure 4:**
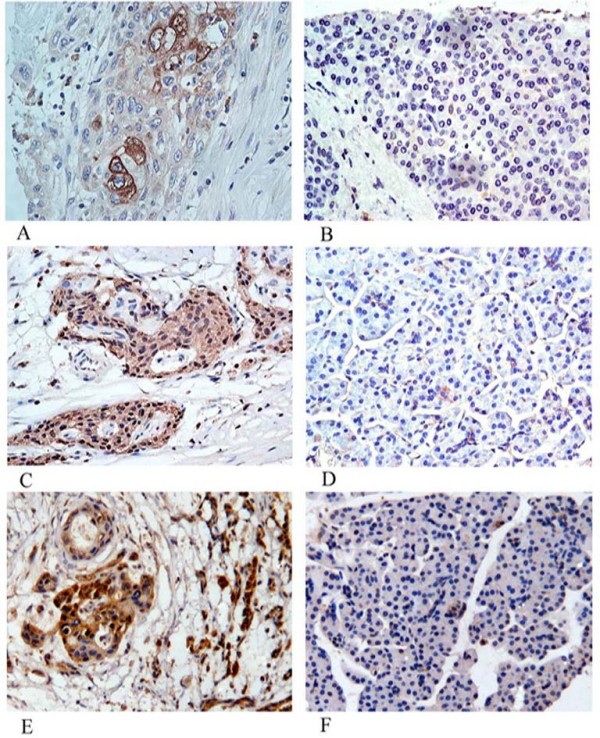
**MMP-9, DJ-1 and A1BG expression analysis by Immunohistochemistry (×200)**. MMP-9 (A), DJ-1 (C) and A1BG (E) over-expressed in PDAC tissues. MMP-9 (B), DJ-1(D) and A1BG (F) were not detectable in normal pancreas tissues.

The expressions of MMP-9, DJ-1 and A1BG were confirmed by immunohistochemistry in 51 pancreatic cancer and 8 normal pancreas samples that were not included in the proteomic experiment (Table 4; Figure 4). MMP-9 was detected in the malignant ductal epithelia in 82.4% of PDAC tissues, but was barely detectable in normal pancreas. In addition, 9 cases of PDAC demonstrated strong MMP-9 expression in the stroma.

**Table 4:** Summary of MMP-9, DJ-1 and A1BG immunohistochemical study on PDAC tissue array

PDAC	Negative			Positive			Total
		
	MMP-9	DJ-1	A1BG	MMP-9	DJ-1	A1BG	
Well differentiated	2 (22.2%)	2 (22.2%)	1 (11.1%)	7 (77.8%)	7 (77.8%)	8 (88.9%)	9
Moderately differentiated	2 (10.5%)	3 (15.8%)	2 (10.5%)	17 (89.5%)	16 (84.2%)	17 (89.5%)	19
Poorly differentiated	5 (21.7%)	9 (39.1%)	4 (17.4%)	18 (78.3%)	14 (60.9%)	19 (82.6%)	23

In normal pancreas, DJ-1 was negatively or weakly expressed in duct epithelium, acinar cells and islet cells. In PDAC, 72.5% demonstrated DJ-1 over-expression in cancer cells. In addition, DJ-1 over-expression was related to the differentiation of PDAC. DJ-1 positive stain was observed in 7/9 well differentiated tumors, 16/19 moderately differentiated tumors, and 14/23 poorly differentiated tumors, respectively. There was a significant difference of DJ-1 over-expression between moderately differentiated PDAC with poorly differentiated PDAC (p < 0.05).

No positive A1BG staining was detected in all the normal pancreas tissues. A1BG was over-expressed in the cytoplasma of malignant epithelia in 86.3% of pancreatic cancer tissues, significantly higher than that in normal pancreas tissues (p < 0.01).

### Serum MMP-9 levels by ELISA

**Figure 5:**
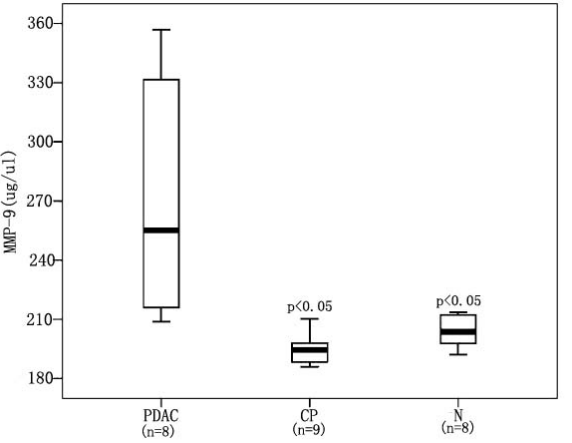
**Box plot of MMP-9 serum levels for PDAC, chronic pancreatitis and normal controls by ELISA**. The serum levels of MMP-9 in PDAC patients were significantly higher than those in chronic pancreatitis patients and in healthy controls (p < 0.05). PDAC: pancreatic ductal adenocarcinoma patients; CP: chronic pancreatitis patients; N: healthy controls.

As shown in Figure 5, serum MMP-9 levels were significantly higher in patients with pancreatic cancer (median = 255.14 ng/ml; quartile range, 125.43 ng/ml) than those with chronic pancreatitis (median = 210.22 ng/ml; quartile range, 12.48 ng/ml; p = 0.009) and normal controls (median = 203.77 ng/ml; quartile range, 17.04 ng/ml; p = 0.027).

## Discussion

PDAC is the most common pathological subgroup of pancreatic cancer. During the development of PDAC, malignant ductal cells preferentially shed into the ductal lumen, making pancreatic juice a rich source of cancer-specific proteins. Therefore, pancreatic juice is an ideal specimen for identifying new tumor markers for PDAC.

We adopted a quantitative proteomic technology (DIGE) to compare the protein profiling of pancreatic juice from PDAC and its cancer-free controls. DIGE technology not only provided reliable quantification, but also can minimize the run-to-run reproducibility of conventional 2-DE [8]. In this study, a total of 24 differentially expressed proteins between pancreatic juice from PDAC and cancer-free controls, including 14 up-regulated proteins and 10 down-regulated proteins, were identified. Half of the up-regulated proteins in PDAC, such as DJ-1, MMP-9, apolipoprotein A-I, A1BG, SOD [Mn], serotransferrin and Ig lambda chain V-IV region were firstly identified in cancerous pancreatic juice. Three up-regulated proteins MMP-9, DJ-1 and A1BG in PDAC pancreatic juice were further validated. Western blot analysis demonstrated elevated protein levels of MMP-9, DJ-1 and A1BG in cancerous pancreatic juice compared with cancer-free pancreatic juice, which is consistent with our proteomic findings.

For the scarcity of cancer-free pancreatic juice specimens, two types of pancreatic juice control subjects, one with pancreatitis and the other without pancreatitis, were included in our study. In a previous proteomic study by Shen et al. [9], it was demonstrated that there are obvious differences in protein profiling between pancreatitis and healthy control tissues. So the protein profiling of pancreatic juice between pancreatitis patients and control subjects without pancreatitis was also compared (Table 3). Seven pancreatic juice proteins, such as trypsin-1 precursor, were demonstrated to be differently expressed between cancer-free controls with and without pancreatitis. In addition, five down-regulated proteins identified in pancreatic cancer juice (carboxypeptidase A2, chymotrypsinogen B, elastase-3A, elastase-3B and trypsin-1) was also down-regulated in pancreatic cancer tissues compared with pancreatitis and normal pancreas tissues as described by Shen et al. [9].

Over-expression of MMP-9 and DJ-1 in pancreatic cancer tissues have been shown in previous studies [10,11]. It is believed that MMP-9 over-expression results in the degradation of the basement membrane and contributes to local invasion or distant metastases during pancreatic carcinogenesis [12,13]. DJ-1 is a novel mitogen-dependent oncogene involved in ras-related signal transduction pathway [14]. Recently, several previous studies have shown that DJ-1 is over-expressed in multiple cancer tissues including pancreatic cancer [15,16]. Our immunohistological study extended the above investigation to larger sample size and revealed that MMP-9 and DJ-1 were expressed in 82.4% and 72.5% of PDAC tissues, significantly higher than that in normal pancreas. Furthermore, DJ-1 over-expression was associated with PDAC differentiation. Moderately differentiated PDAC demonstrated a higher expression level of DJ-1 than poorly differentiated tumor subgroup, which suggests that DJ-1 may be a differentiation related protein.

A1BG, a member of the immunoglobulin superfamily, is believed to be a secreted plasma protein, but its function is unknown [17]. In this study, we first identified and confirmed A1BG as an elevated protein in pancreatic juice and cancer tissues of PDAC. Our immunohistochemical study further validated that A1BG protein over-expression was seen in most PDAC tissues, but not detected in normal pancreas tissues. Using a glycoprotein profiling method, Kreunin et al. [18] recently found that A1BG was detected in urinary samples from bladder cancer patients, but in none of the samples obtained from non-tumor-bearing individuals. Yoon et al. [19] also demonstrated A1BG over-expression in liver cancer cell lines and tissues by semi-quantitative RT-PCR. These findings together with our proteomic results suggest that A1BG might be a cancer-associated gene and a novel tumor marker of cancer, and its possible functions in carcinogenesis deserve further investigation.

The novel protein biomarkers identified in ERCP-obtained pancreatic juice might have enormous potential for use in the diagnosis of PDAC. It is reasonable to believe that proteins indicative of cancer in pancreatic juice are also elevated in blood. Therefore, we measured serum levels of MMP-9 in PDAC, and found that serum MMP-9 had a higher level in PDAC than in chronic pancreatitis and healthy controls. The findings suggest that serum measurement of MMP-9 or other up-regulated proteins in cancerous pancreatic juice might be helpful in the diagnosis of pancreatic adenocarcinoma and deserve further investigation.

## Conclusion

In the present study, we carried out a comparative proteomics analysis on pancreatic juice from PDAC and its cancer-free controls. A total of 24 differentially expressed proteins were identified, of which 14 were over-regulated and 10 were under-regulated in pancreatic juice of PDAC. We further confirmed the expression levels of three up-regulated proteins (MMP-9, DJ-1 and A1BG) in pancreatic juice, tissue and serum samples using Western blot, IHC or ELISA. A1BG was firstly identified as a biomarker in cancer-associated samples. Serum MMP-9 measurement might be helpful in discriminating pancreatic adenocarcinoma from chronic pancreatitis and healthy controls. The newly identified proteins in this study might be useful for developing new pancreatic juice-related or serum-related diagnostic markers for PDAC.

## Competing interests

The authors declare that they have no competing interests.

## Authors' contributions

MT and YZC participated in the experimental design, electrophoresis, trypsin digestion, MS identification, Western blot, statistical analysis and the manuscript writing; JXH was responsible for the experimental design, technique and financial support, and manuscript writing; GHS and XYZ were responsible for IHC experiments; MJZ and YC participated in electrophoresis, trypsin digestion. All authors contributed to this work read and approved the final manuscript.

## Pre-publication history

The pre-publication history for this paper can be accessed here:


